# Supplementary material for: Identification and Quantification of a Phytotoxic Metabolite from Alternaria dauci
Source: Molecules. 2020 Sep 2;25(17):4003. doi: 10.3390/molecules25174003 (PMC7504779; doi:10.3390/molecules25174003)
Supplement: Supplementary file 1 [file molecules-25-04003-s001.pdf]

Supplementary material

Identification and quantification of a phytotoxic metabolites from *Alternaria dauci*

Martha Leyte-Lugo [1,*], Pascal Richomme [2], Pascal Poupard [3] and Luis M. Peña-Rodriguez [1,*]

[1] Unidad de Biotecnología, Centro de Investigación Científica de Yucatán, 97205, Mérida, Yucatán, Mexico
[2] UPRES EA921SONAS, SFR 4207 QUASAV, Université d'Angers, Angers, 49045, France; pascal.richomme@univ-angers.fr
[3] UMR 1345 IRHS, SFR 4207 QUASAV, INRAE, Institut Agro, Université d'Angers, Angers, 49045, France; pascal.poupard@univ-angers.fr
* Correspondence: lmanuel@cicy.mx (L.M.P-R.); mleyte@correo.xoc.uam.mx (M.L.-L.); Tel.: +52-9998-990767(L.M.P-R.); Tel.: +52-5529-631088 (M.L.-L.)

Academic Editor: Francisco Leon
Received: 20 August 2020; Accepted: 1 September 2020; Published: date

Table of contents

	Page
**Table S1**. Regression equations, linear ranges, LOQs, LODs, and precision for quantitative analysis.	2
**Table S2.** Average recovery of the phytotoxins quantified in *A. dauci* organic fractions.	2
**Figure S1**. Production kinetics of **a**) α-acetylorcinol (**1**) and **b**) *p*-hydroxybenzoic acid (**2**) in cultures of *A. dauci*.	3
**Figure S2**. ^{1}H NMR spectrum (600 MHz, CD_3OD) of α-acetylorcinol (**1**).	3
**Figure S3**. ^{13}C NMR spectrum (150 MHz, CD_3OD) of α-acetylorcinol (**1**).	4
**Figure S4**. IR spectrum of α-acetylorcinol (**1**).	4
**Figure S5**. ^{1}H NMR spectrum (600 MHz, CD_3OD) of *p*-hydroxybenzoic acid (**2**).	5
**Figure S6**. ^{13}C NMR spectrum (150 MHz, CD_3OD) of *p*-hydroxybenzoic acid (**2**).	5
**Figure S7**. IR spectrum of *p*-hydroxybenzoic acid (**2**).	6
**Figure S8**. ^{1}H NMR spectrum (600 MHz, $CDCl_3$) of O,O-dimethyl-α-acetylorcinol.	6
**Figure S9**. IR spectrum of O,O-dimethyl-α-acetylorcinol.	7

Table S1. Regression equations, linear ranges, LOQs, LODs, and precision for quantitative analysis.

Phytotoxin	Regression equation[a]	Linear range (mg/mL)	r^2	LOQ (mg/mL)	LOD (mg/mL)	Precision RSD[b] (%)	
						Intraday	Interday
α-acetylorcinol	y = 70806095.0x − 3993858.9	0.1 – 0.5	0.9966	0.05	0.02	0.83	0.58
p-hydroxybenzoic acid	y = 62420155.9x − 1823036.0	0.05 – 0.5	0.9971	0.04	0.01	1.68	0.87

[a]In the regression equation y = mx + b, x refers to the concentration of standards (mg/mL), y the peak area.
[b]RSD (%) = (SD/mean) × 100
r^2 is the correlation coefficient; LOD is the limit of detection; LOQ, is the limit of quantification.

Table S2. Average recovery of the phytotoxins quantified in *A. dauci* organic fractions.

Phytotoxin	Amount added (mg/mL)	Amount found (mg/mL)	Average recovery (%)	RSD[a] (%)
α-acetylorcinol	0.20	0.20	99.67	0.92
	0.30	0.30	100.21	1.46
	0.40	0.40	100.95	0.32
p-hydroxybenzoic acid	0.05	0.05	100.77	1.10
	0.10	0.10	100.91	0.66
	0.20	0.20	99.54	1.05

[a]RSD (%) = (SD/mean) × 100.

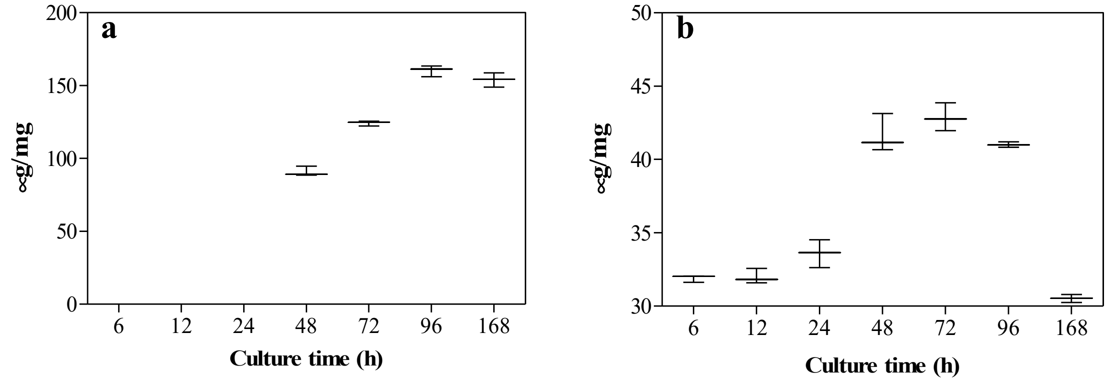

Figure S1. Production kinetics of **a**) α-acetylorcinol (**1**) and **b**) *p*-hydroxybenzoic acid (**2**) in cultures of *A. dauci*.

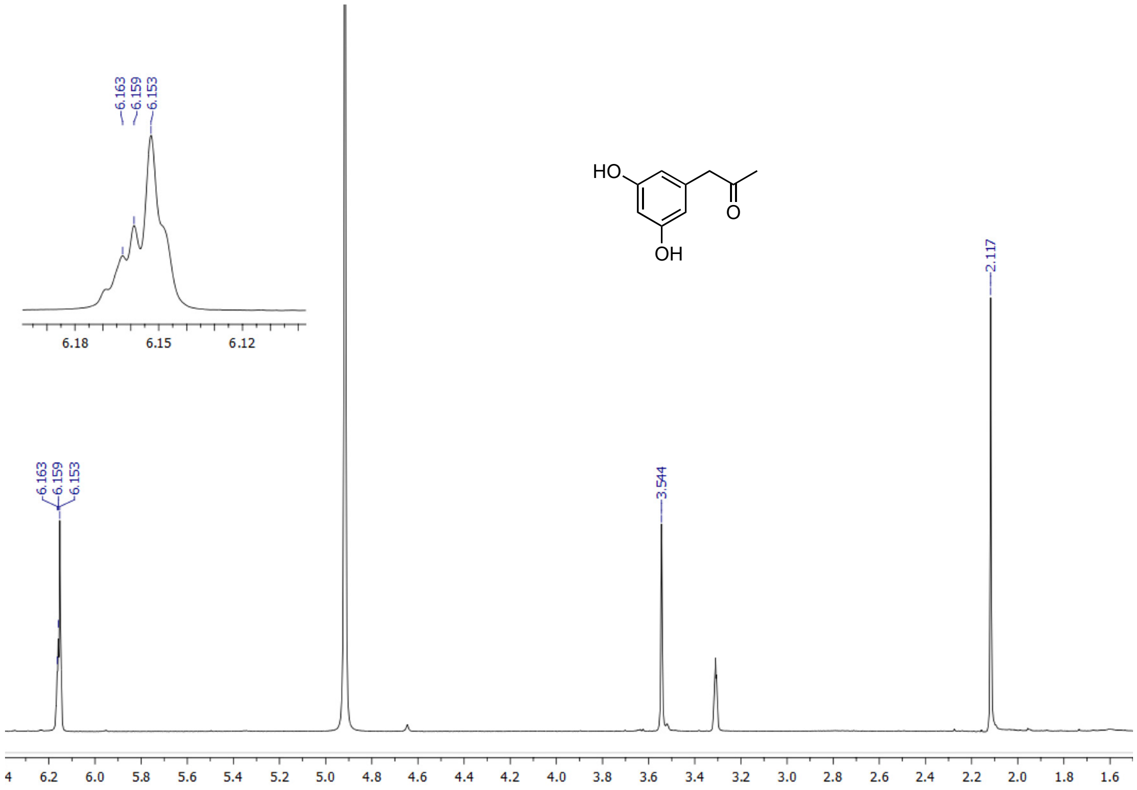

Figure S2. ^{1}H NMR spectrum (600 MHz, CD_3OD) of α-acetylorcinol (**1**).

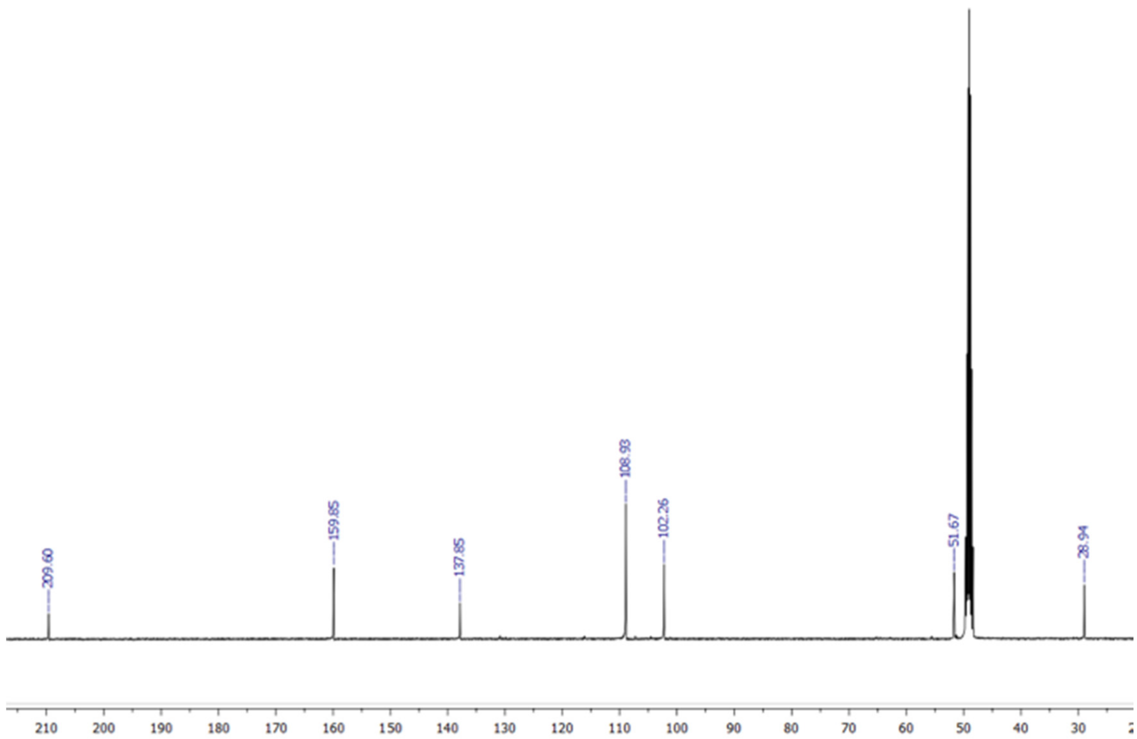

Figure S3. ^{13}C NMR spectrum (150 MHz, CD_3OD) of α-acetylorcinol (**1**).

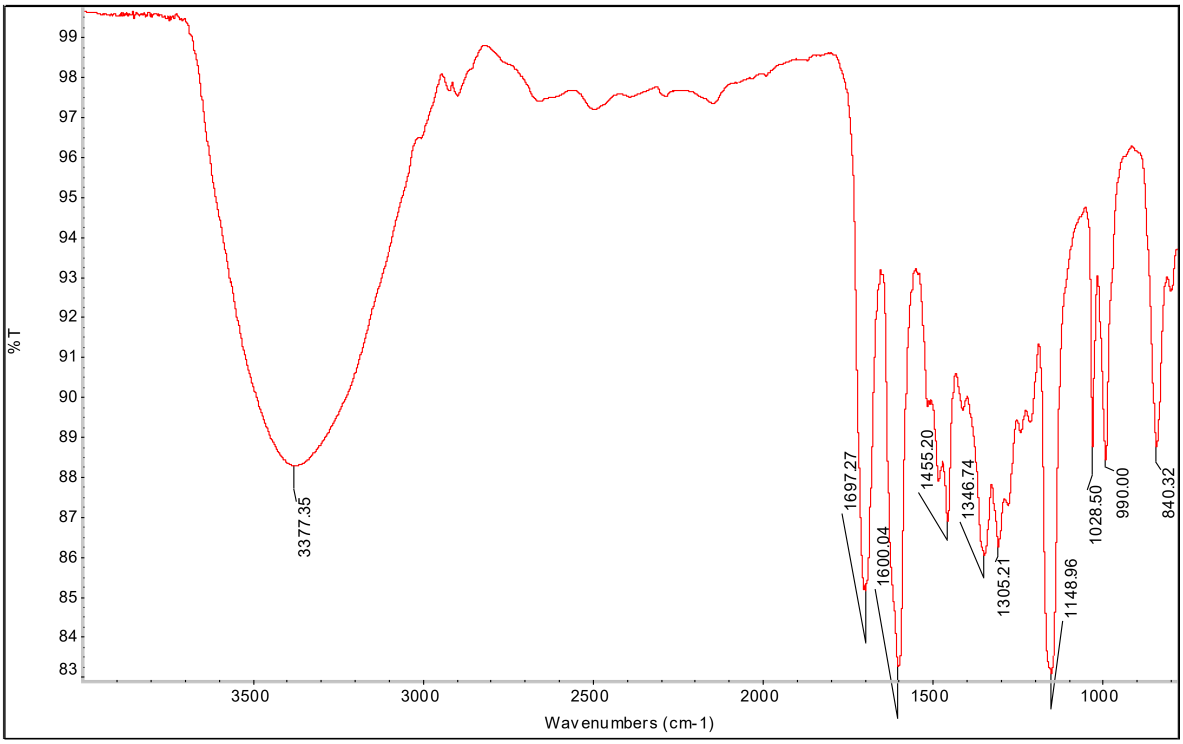

Figure S4. IR spectrum of α-acetylorcinol (**1**).

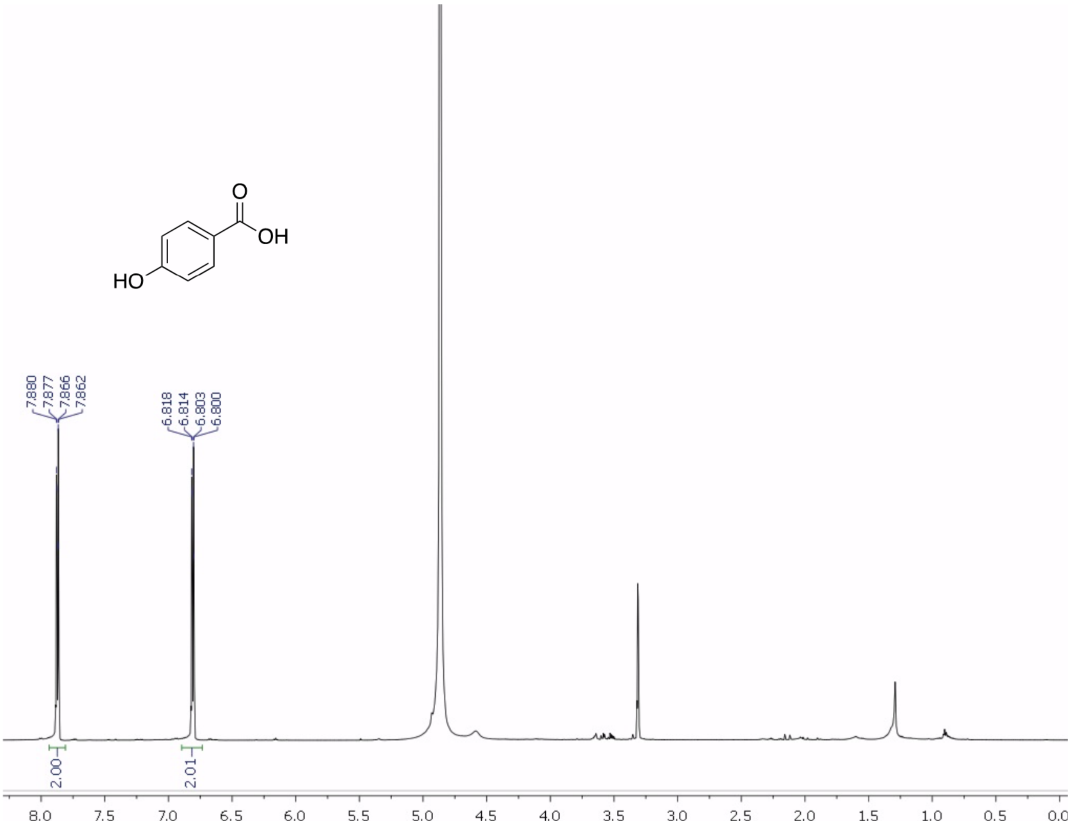

Figure S5. ^{1}H NMR spectrum (600 MHz, CD_3OD) of *p*-hydroxybenzoic acid (**2**).

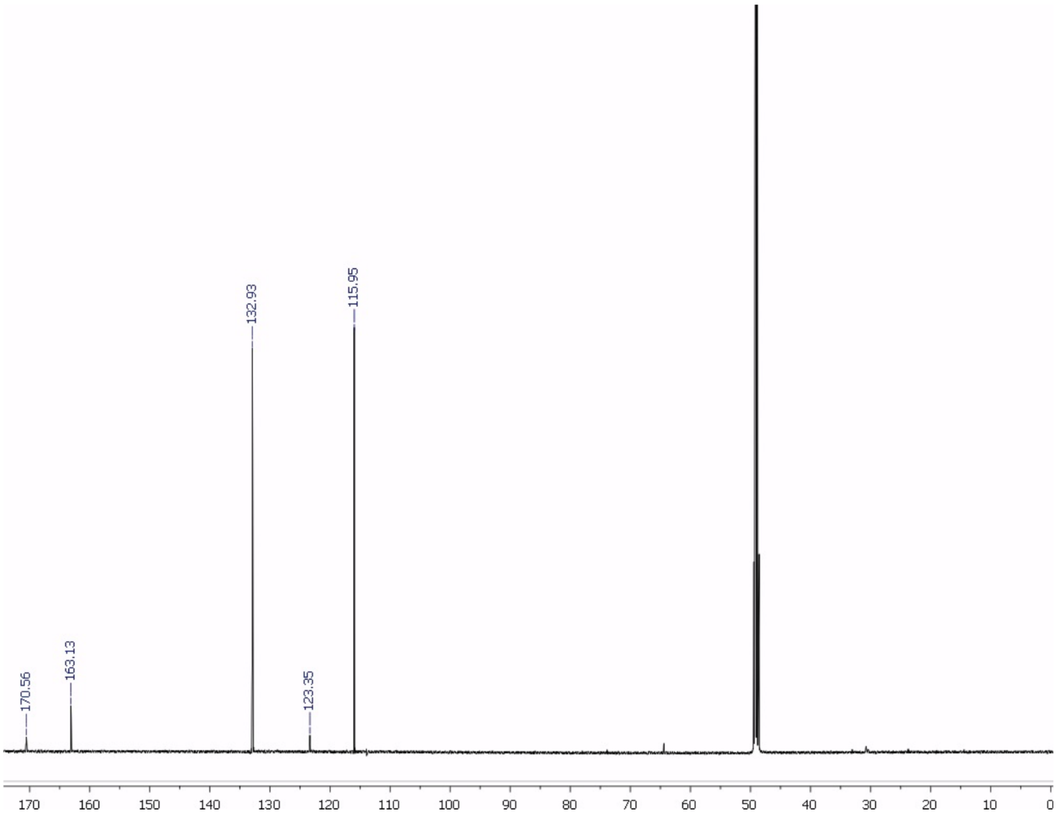

Figure S6. ^{13}C NMR spectrum (150 MHz, CD_3OD) of *p*-hydroxybenzoic acid (**2**).

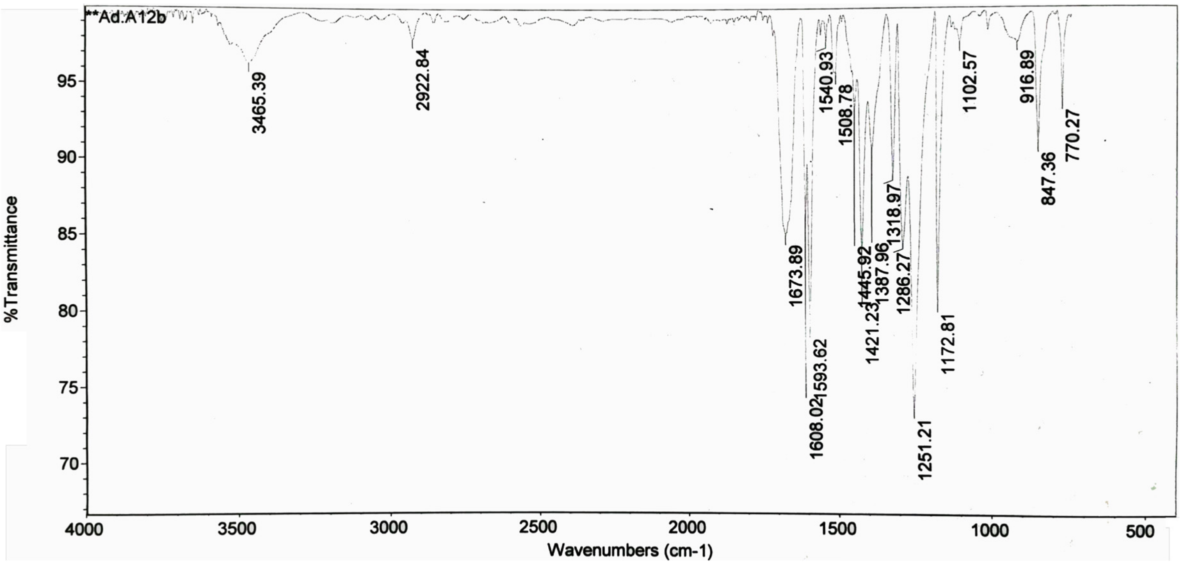

Figure S7. IR spectrum of *p*-hydroxybenzoic acid (**2**).

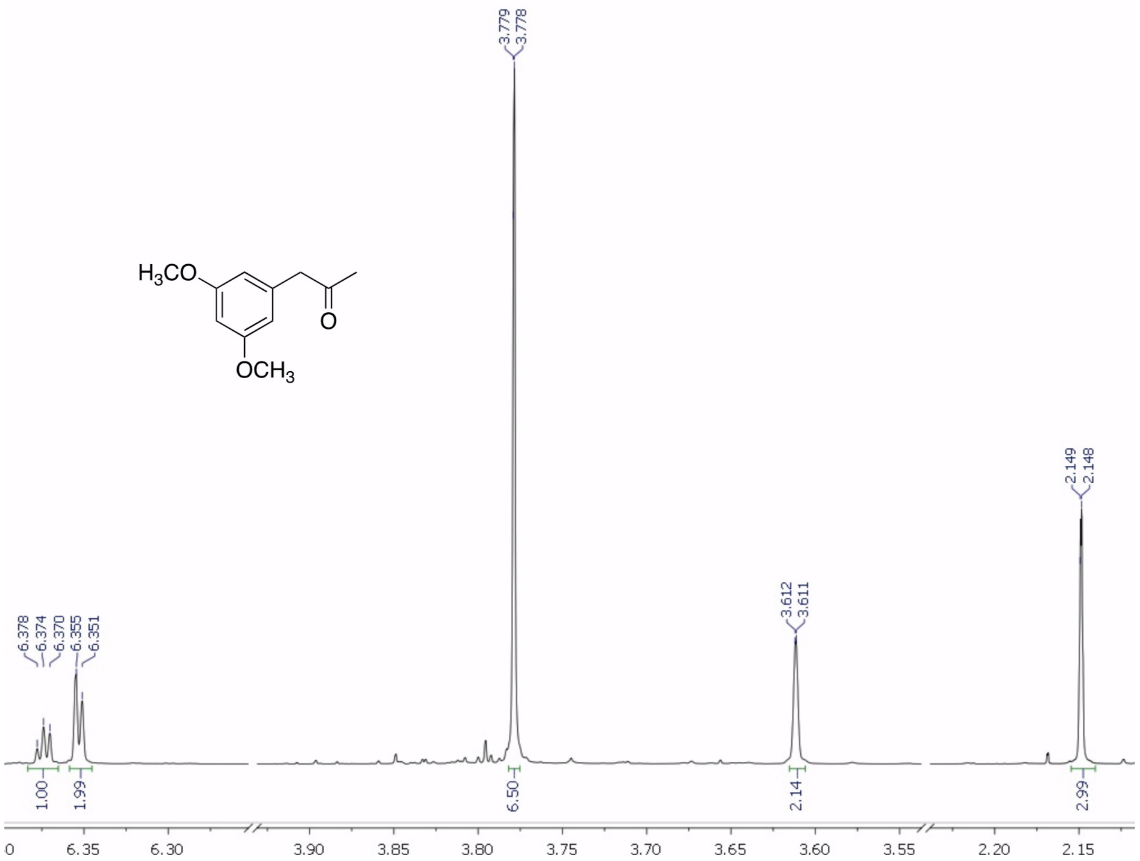

Figure S8. ^1H NMR spectrum (600 MHz, $CDCl_3$) of O,O-dimethyl-α-acetylorcinol.

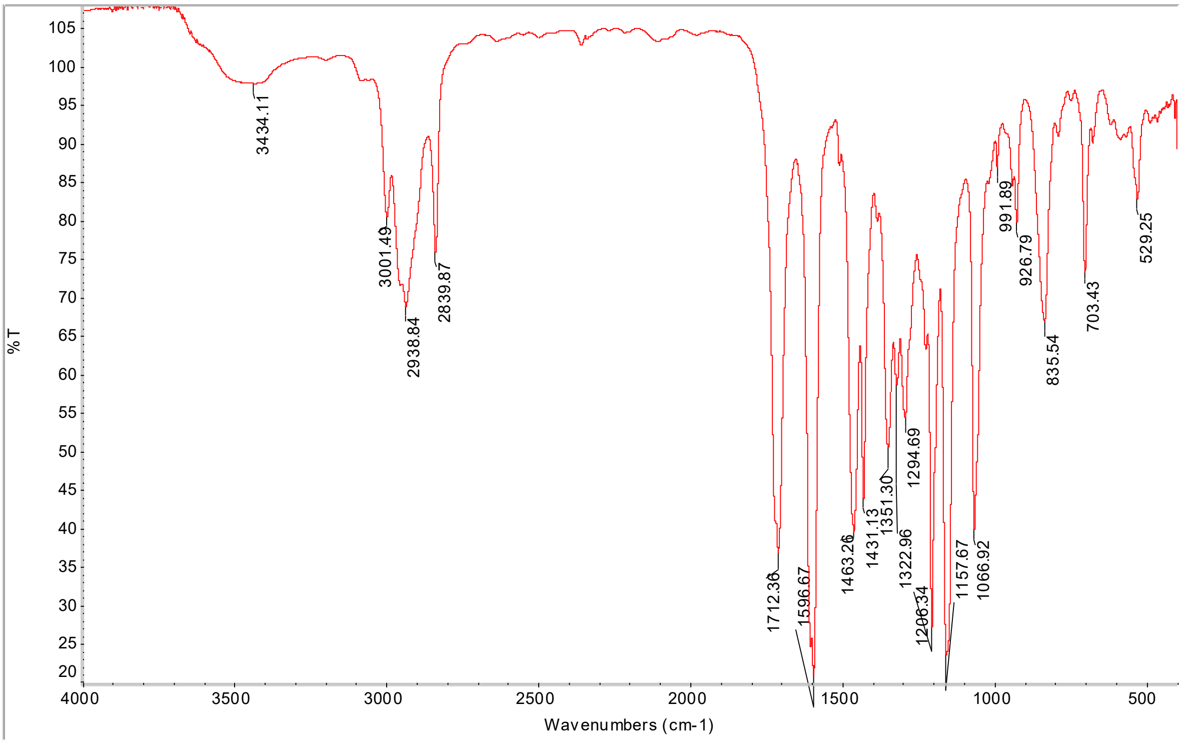

Figure S9. IR spectrum of O,O-dimethyl-α-acetylorcinol.
